# Descriptive epidemiology and phylogenetic analysis of highly pathogenic avian influenza H5N1 clade 2.3.4.4b in British Columbia (B.C.) and the Yukon, Canada, September 2022 to June 2023

**DOI:** 10.1080/22221751.2024.2392667

**Published:** 2024-08-15

**Authors:** Shannon L. Russell, Cassandra L. Andrew, Kevin C. Yang, Michelle Coombe, Glenna McGregor, Tony Redford, Agatha N. Jassem, James E. A. Zlosnik, Jolene Giacinti, Kevin S. Kuchinski, John L. Palmer, John R. Tyson, Chris Fjell, Megan Willie, Megan V. Ross, Maeve Winchester, Laurie Wilson, Yohannes Berhane, Caeley Thacker, N. Jane Harms, Catherine Soos, Theresa Burns, Natalie Prystajecky, Chelsea Himsworth

**Affiliations:** a British Columbia Centre for Disease Control (BCCDC) Public Health Laboratory, Vancouver, Canada; b Department of Pathology and Laboratory Medicine, University of British Columbia, Vancouver, Canada; c School of Population and Public Health, Faculty of Medicine, University of British Columbia, Vancouver, Canada; d Public Health Agency of Canada (PHAC), Winnipeg, Canada; e Animal Health Centre, British Columbia Ministry of Agriculture and Food, Abbotsford, Canada; f Ecotoxicology and Wildlife Health Division, Environment and Climate Change Canada (ECCC), Ottawa, Canada; g Canadian Wildlife Service, Environment and Climate Change Canada (ECCC), Delta, Canada; h British Columbia Ministry of Water, Land and Resource Stewardship, Nanaimo, Canada; i Canadian Food Inspection Agency, Winnipeg, Canada; j Department of Environment, Government of Yukon, Whitehorse, Canada; k Canadian Wildlife Health Cooperative British Columbia, Abbotsford, Canada

**Keywords:** Avian influenza, clade 2.3.4.4b, HPAI, H5N1, molecular epidemiology, phylodynamics, surveillance, whole genome sequencing

## Abstract

Surveillance data from wildlife and poultry was used to describe the spread of highly pathogenic avian influenza (HPAI) H5N1 clade 2.3.4.4b in British Columbia (B.C.) and the Yukon, Canada from September 2022 – June 2023 compared to the first “wave” of the outbreak in this region, which occurred April – August 2022, after the initial viral introduction. Although the number of HPAI-positive poultry farms and wildlife samples was greater in “Wave 2”, cases were more tightly clustered in southwestern B.C. and the most commonly affected species differed, likely due to an influx of overwintering waterfowl in the area. Eight HPAI genetic clusters, representing seven genotypes and two inter-continental viral incursions, were detected, with significant variation in the relative abundance of each cluster between the waves. Phylogenetic data suggests multiple spillover events from wild birds to poultry and mammals but could not rule out transmission among farms and among mammals.

## Introduction

Highly-pathogenic avian influenza virus (HPAIV) is a globally distributed pathogen that can cause significant morbidity and mortality in domestic poultry [1,2]. During 2021–2023, North America suffered from the worst avian influenza virus (AIV) epizootic event in the history of the continent. As of August 2023, HPAIV H5N1 clade 2.3.4.4b had affected more than 1000 farms and 65 million domestic birds in 47 American states and nine Canadian provinces [3,4].

Wild birds, particularly waterfowl, are the reservoir for AIV, spreading the virus between jurisdictions during migration [5]. Surveillance for HPAI in wild birds can be used to understand the epidemiology of these viruses in nature and the risk of spillover into poultry [6]. Recently, H5N1 clade 2.3.4.4b has demonstrated the ability to infect mammals, particularly mesocarnivores who consume infected wild birds [7]. These infections have raised concerns that the virus may adapt to mammals and spillover into humans [8].

HPAI H5N1 spread to poultry in the province of British Columbia (B.C.), Canada in April 2022, likely in association with the northward spring migration of infected wild birds from the USA via the Pacific flyway [9]. Indeed, HPAI-related wildlife mortality was detected throughout B.C. and the Yukon. Subsequently, a total of 19 farms were affected before the apparent end of the outbreak in poultry in mid-June 2022. Wildlife surveillance, however, demonstrated continued circulation of the virus in wild birds in B.C. throughout the summer, albeit at low prevalence [9]. In mid-September 2022, a second, more explosive “wave” of HPAI H5N1 spread through both wild birds and poultry, affecting 85 farms between September 12, 2022, and January 21, 2023. The objective of this study was: 1) to describe the findings from wild bird surveillance during the second wave of HPAI H5N1 in B.C. and the Yukon; 2) to compare lineages circulating in wildlife to those infecting poultry; 3) to describe spillover into wild mammals in the province/territory during this timeframe; and 4) to identify similarities and differences between the first and second waves of HPAI H5N1 in this region.

## Materials and methods

### Sample and data collection

In B.C., sick and dead wild birds and mammals were reported to and collected by the B.C. Ministry of Water, Land and Resource Stewardship (WLRS) or to the Canadian Wildlife Service (CWS) – Environment and Climate Change Canada (ECCC) and subsequently submitted to the Ministry of Agriculture and Food (MAF) Animal Health Centre (AHC), in Abbotsford, B.C., where oropharyngeal/nasopharyngeal and rectal/cloacal swabs were obtained. In the Yukon, sick and dead wild birds and mammals were reported to and collected by wildlife agency officials or the Animal Health Unit (AHU) in the Government of Yukon Department of Environment. Oropharyngeal/nasopharyngeal swabs were collected by Animal Health Unit staff. In B.C., CWS also obtained oral and cloacal swabs from waterfowl collected lethally during permitted activities (Supplementary Material) and hunter-harvested waterfowl, as well as from live surf scoters (*Melanitta perspicillata*) and white-winged scoters (*Melanitta deglandi*) captured as part of sea duck monitoring activities. Oropharyngeal and cloacal swabs from poultry were collected by the Canadian Food Inspection Agency (CFIA) upon first suspicion of an outbreak.

Swabs were sent to the B.C. Centre for Disease Control (BCCDC; wild animals) or the AHC (poultry) for testing. Data collected included location and date of carcass collection or sampling (for live birds and poultry), as well as species and poultry production type [3].

### AIV PCR, subtyping and sequencing

At the BCCDC, wildlife samples were screened for AIV using a quantitative reverse-transcription polymerase chain reaction (qRT-PCR) assay targeting a conserved region in the matrix (M) gene [10], then subtyped for H5 using an in-house designed BCCDC lab-developed test (LDT) targeting the hemagglutinin (HA) gene segment of influenza A virus (IAV) [11]. Matrix-positive samples (Ct <36), regardless of subtype, were submitted for whole genome sequencing (WGS). While all matrix-positive samples were sequenced, non-H5 subtypes were excluded from phylogenetic analyses.

WGS libraries were generated as previously described [12–15]. WGS analysis was performed using the Nextflow pipeline FluViewer_nf v0.2.0 [16]. Consensus sequences were generated by aligning reads (bwa v0.7.17) to a curated reference database. HA and neuraminidase (NA) subtypes were determined based on the subtype associated with the best match in the reference database. *In silico* HPAIV pathotyping was also performed [17] to identify the H5 HPAI motif PLREKRRKRGLF in the HA cleavage site. For inclusion in the phylogenetic analysis, sequences required a minimum 20X depth and 90% coverage across each segment.

At the AHC, poultry samples were screened for AIV using an IAV M gene qRT-PCR assay, followed by the H5 subtyping assay [18], and positive samples (Ct <36) were submitted to the National Centre for Foreign Animal Disease (NCFAD) for WGS as previously described [19]. The NCFAD provided one sequence per infected farm for this analysis.

### Data analysis

Animals tested between September 12, 2022, and June 16, 2023, were included in this study. A wild animal or poultry flock was considered HPAI H5N1-positive if they tested positive for IAV by qRT-PCR and were identified as H5 positive either by subtyping qRT-PCR and/or WGS. No H5 viruses other than HPAI H5N1 clade 2.3.4.4b were detected, therefore samples that were H5-positive but failed sequencing (Ct values >30 were more likely to fail WGS) were presumed to be HPAI H5N1. Descriptive epidemiological analyses were performed in R (v4.2.1 GUI 1.79 High Sierra build (8095)) [20] and ArcGIS Pro (v3.1.2 2023). SaTScan™ (v10.1 2023) was used to separately identify geographic clustering of wild and domestic birds infected with genetically related viruses (Supplementary Materials).

Phylogenetic trees (based on nucleotide sequences) for all segments were constructed using fluflo (v0.1.3) (https://github.com/BCCDC-PHL/fluflo), a Nextflow wrapper for Nextstrain containing IQTREE (v2.1.4_beta) and visualized using Nextstrain (v13.0.0)/Auspice (v2.40.0). Additionally, the H5Nx genotyping tool GenoFLU [21] was used to assign genotypes to all segments of the sequenced HPAI H5N1 genomes. Non-H5 AIV sequences (*N *= 12) were also analyzed using the GenoFLU pipeline to determine if any of the genotype assignments for the internal segments overlapped with the B.C./Yukon HPAI H5N1 dataset. Detailed methods on HPAIV sequence analysis can be found in the Supplementary Materials Technical Appendix.

## Results

### Descriptive epidemiology of HPAI H5N1 in wildlife and poultry in B.C. and the Yukon and differences between two outbreak “waves’

In “Wave 2”, the B.C. passive surveillance programme collected 394 wild bird samples representing 67 different species, of which 134 (34%) were positive for HPAI H5N1 (Table 1 and Supplementary Table 1). From the Yukon, 23 avian samples were tested, representing 14 different species, of which two (9%) were positive (Supplementary Table 1). The relative frequency of detections in different wild bird species varied between the two waves. Specifically, compared to “Wave 1” there was an increase the diversity of infected avian taxa detected in “Wave 2” (e.g. infected cackling geese (*Branta hutchinsii*) were only detected in “Wave 2”), and, for those taxa infected in both waves, the number of detections was generally greater in “Wave 2”, with the exception of Canada geese (*Branta canadensis*) and Bald eagles (*Haliaeetus lucocephalus*) (Table 1).

**Table 1.** Case counts for the top 15 HPAI H5N1-positive wild animal species from British Columbia (B.C.) and the Yukon Territory (YT) for “Wave 1” (April 12 – September 11, 2022) and “Wave 2” (September 12, 2022 – June 13, 2023).

Species	Case count “Wave 1”	Case count “Wave 2”
American crow (*Corvus brachyrhynchos*)^a^	3	16
Canada goose (*Branta canadensis*)	47^b^	15
Trumpeter swan (*Cygnus buccinator*)	1^b^	15
Lesser snow goose (*Anser caerulescens*)	5^b^	14
Striped skunk (*Mephitis mephitis*)	1	14
Red-tailed hawk (*Buteo jamaicensis*)	1	13
Great horned owl (*Bubo virginianus*)	12	12
Bald eagle (*Haliaeetus leucocephalus*)	25^b^	7^b^
Cackling goose (*Branta hutchinsii*)	0	7
Mallard (*Anas platyrhynchos*)	2	7^c^
Peregrine falcon (*Falco peregrinus*)	2	6
Barn owl (*Tyto alba*)	1	4
Cooper's hawk (*Accipiter cooperii*)	2	4
Glaucous-winged gull (*Larus glaucescens*)	1	4
Great blue heron (*Ardea herodias*)	5	3

^a^ Likely incorrectly classified as a northwestern crow in “Wave 1”.

^b^ From “Wave 1”: two Canada geese, one trumpeter swan, one snow goose, and three bald eagles were from YT. From Wave 2: one bald eagle was from the Yukon..

^c^ All positive mallards from Wave 2 were from active – hunter harvested surveillance.

In B.C., 87 samples from hunter harvested birds representing six species were collected of which 10 (11.5%) were HPAI H5N1-positive (Supplementary Table 1). A total of 50 birds representing two species were live sampled, of which none tested positive (Supplementary Table 1).

There were 21 wild mammals from seven species that were tested for HPAI H5N1 in B.C. and the Yukon, of which 14 (67%) were positive, all of which were striped skunks (*Mephitis mephitis*) (Supplementary Table 1).

HPAI H5N1 was detected on 85 B.C. poultry farms in “Wave 2”, including 73 (85.9%) commercial and 12 (14.1%) non-commercial farms. There were no domestic poultry detections in the Yukon. In contrast, of the 18 poultry farms affected in “Wave 1”, only four (22.2%) were commercial flocks.

**Figure 1.**
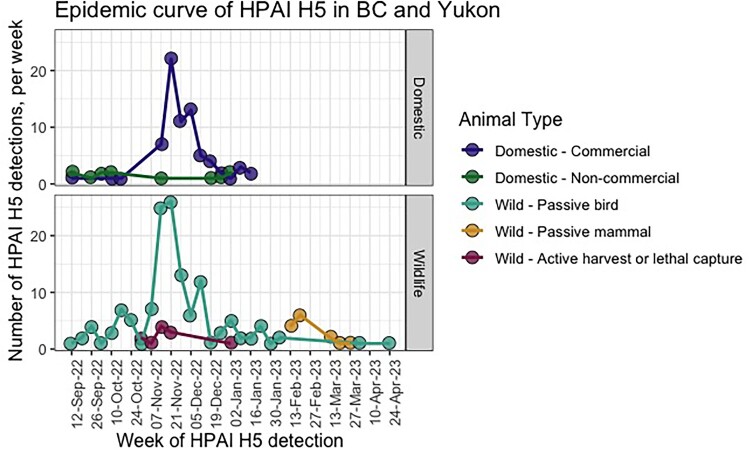
Timeline (epidemic curve) of detections of HPAI H5N1 by qRT-PCR in wildlife, poultry and domestic animals in British Columbia (B.C.) and the Yukon, Canada tested from September 12 – June 16, 2023. There were no positive detections between April 23 and June 16, 2023.

The highest numbers of HPAI H5N1 detections in both wild and domestic birds occurred between November to December 2022, while the wild mammal detections occurred between February and March 2023 (Figure 1).

### Phylogenetic analysis of H5N1 clade 2.3.4.4b viruses detected in wildlife and poultry in B.C. and the Yukon

Of the 160 wildlife samples that were H5-positive by qRT-PCR, 116 generated high-quality HA sequence data (including 83 full and 33 partial genomes), and all were confirmed as HPAI H5N1 clade 2.3.4.4b. HPAI H5N1 sequences were also available from 84 of the 85 affected poultry farms. The HPAI motif in the HA cleavage site was present in all H5N1 sequences.

**Figure 2.**
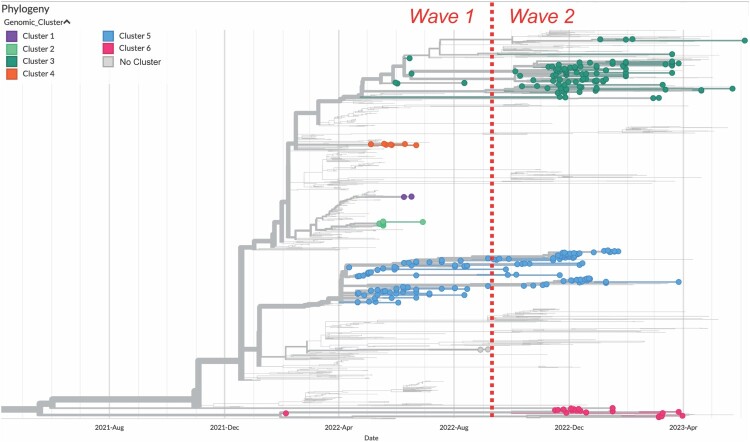
Hemagglutinin (HA)-specific phylogenetic analysis of HPAI H5N1 detections in British Columbia (B.C.) and the Yukon contextualized by H5N1 sequences from other parts of North and South America between September 2021 and July 2023. H5N1 sequences displayed by genetic cluster designation (B.C. and Yukon sequences only), highlighting six genetically distinct HA phylogenies that were in circulation over the course of the outbreak in B.C./Yukon. Sequences are plotted based on specimen collection date. Trees are rooted on the A/Goose/Guangdong/1/96 (*Gs*/*Gd*) (H5) reference sequence.

The presence of two distinct waves of H5N1 infections were evident from phylogenetic analysis, highlighting April – June 2022 and October – December 2022 as periods of peak positivity (Figure 2). These waves were apparent in both local B.C./Yukon data as well as publicly available sequence data from the Americas. HPAI H5N1 viruses detected in B.C./Yukon were closely related to those circulating in the north and southwestern states of the USA during the same time period (Supplementary Figure 1).

A total of six distinct genetic clusters based on HA-specific analysis (denoted Clusters 1-6) were identified between “Wave 1” and “Wave 2”. We defined a genetic cluster as a group of sequences that were subjectively more closely related to each other compared to other sequences in the tree. Genetic relationships among samples in these clusters were maintained across all segments of the genome apart from Clusters 3 and 6, each of which contained two distinct reassortment patterns following full genome analysis (see genotype classification below). Only viruses from Clusters 3, 5 and 6 were detected in both waves. Clusters 1, 2 and 4 were only detected in “Wave 1” (Figure 2). All Clusters were detected in both wildlife and poultry. The most frequently detected Clusters in “Wave 2” were Cluster 3 in wild birds and Cluster 5 in poultry.

**Figure 3.**
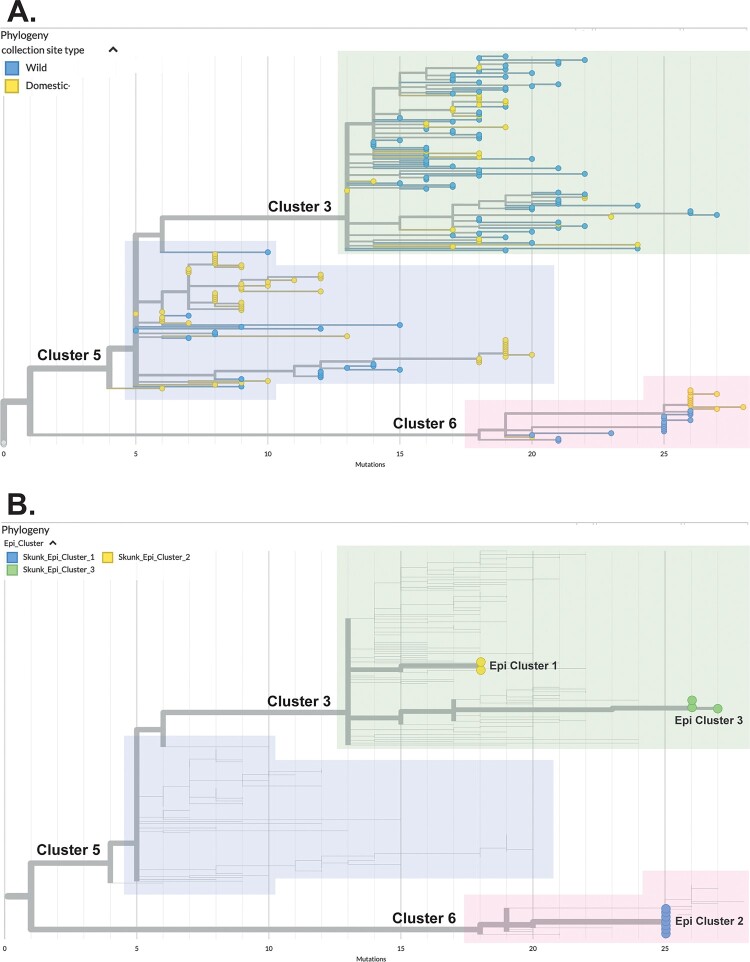
Genetic relationships between domestic, wild bird and mammal sequences among hemagglutinin (HA)-defined genetic clusters suggest multiple modes of transmission contributed to spread during “Wave 2” of the British Columbia (B.C.)/Yukon outbreak. This is demonstrated by A) different collection site type distribution (wild, domestic) among genetic clusters and B) co-clustering of skunk viruses linked spatiotemporally based on associated epidemiological data (denoted by “Epi Cluster” labels). Tree tip labels in (B) have been enlarged for visibility. Trees are rooted on the earliest North American H5N1 detection in the 2021/2022 outbreak, A/chicken/NL/FAV-0033/2021.

Poultry sequences belonging to Cluster 3 were genetically interspersed between wildlife detections (with the exception of one poultry-specific sub-clade identified through full genome analysis), whereas poultry sequences belonging to Clusters 5 and 6 tended to form their own sub-clades, containing few or no closely related wildlife sequences (Figure 3A). Within these poultry sub-clades, highly clonal HA segments were observed, representing viruses from multiple infected farms. These domestic subclades were also observed when other gene segments of the virus were examined (data not shown).

Among the 14 HPAI H5N1-positive striped skunks, which were from three epidemiologically-linked groups (referred to as “Epi Clusters”, Supplementary Figure 2), samples from 12 animals were successfully sequenced. Each of the three groups were infected with genetically distinct H5N1 viruses (Figure 3B); however, animals belonging to the same group were infected with nearly identical viruses. The viruses detected in skunks were related to those circulating in wild and domestic birds at the time.

Mutations associated with HPAI H5N1 adaptation in mammalian hosts were identified in both avian and mammalian HPAIV sequences from B.C./Yukon throughout “Wave 2” of the outbreak (Supplementary Table 2). However, PB2-encoded E627 K (11%), E627A (33%) and D701N (56%), and NP-encoded N319 K (58%) were observed in a disproportionately high number of skunk viruses.

### Temporal and spatial trends in the detection of different HPAI H5N1 clusters among wildlife and poultry in B.C. and the Yukon

**Figure 4.**
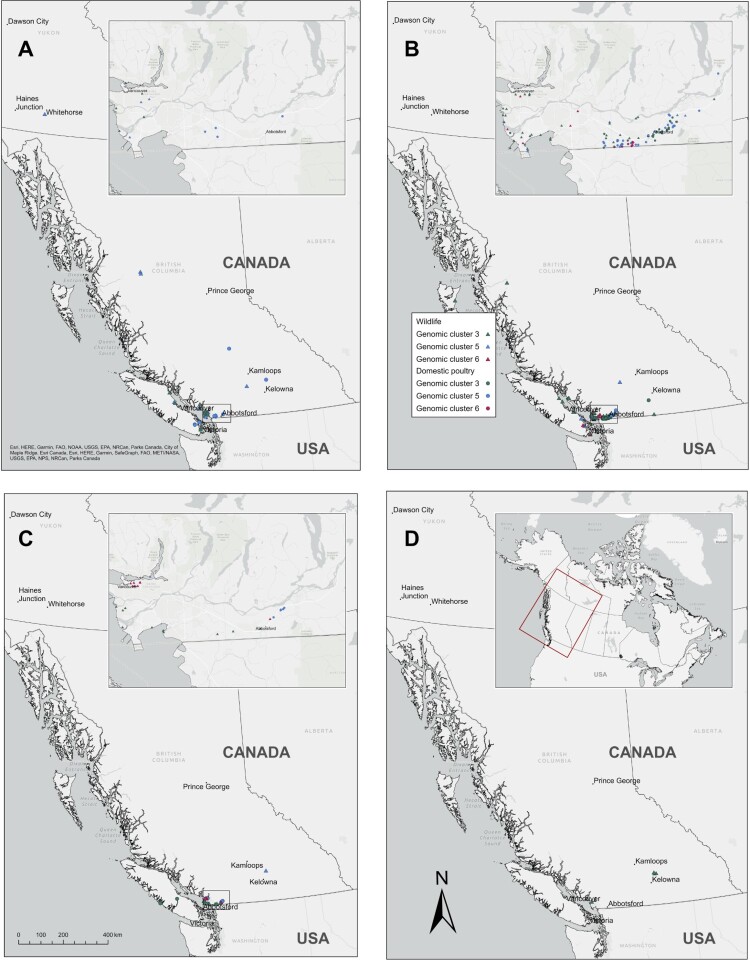
Spatial and temporal distribution of HPAI H5N1 genetic clusters in wildlife and domestic poultry from British Columbia (B.C.) and the Yukon, Canada by death date in A) September to October 2022, B) November to December 2022, C) January to February 2023, and D) March to April 2023. Symbols indicate collection type (wildlife [triangle] or domestic birds [circles]) and colours indicate genomic cluster identification – Cluster 3, green; Cluster 5, blue; and Cluster 6, pink. Inset maps display the area with highest density of detections throughout the outbreak (excluding D).

**Figure 5.**
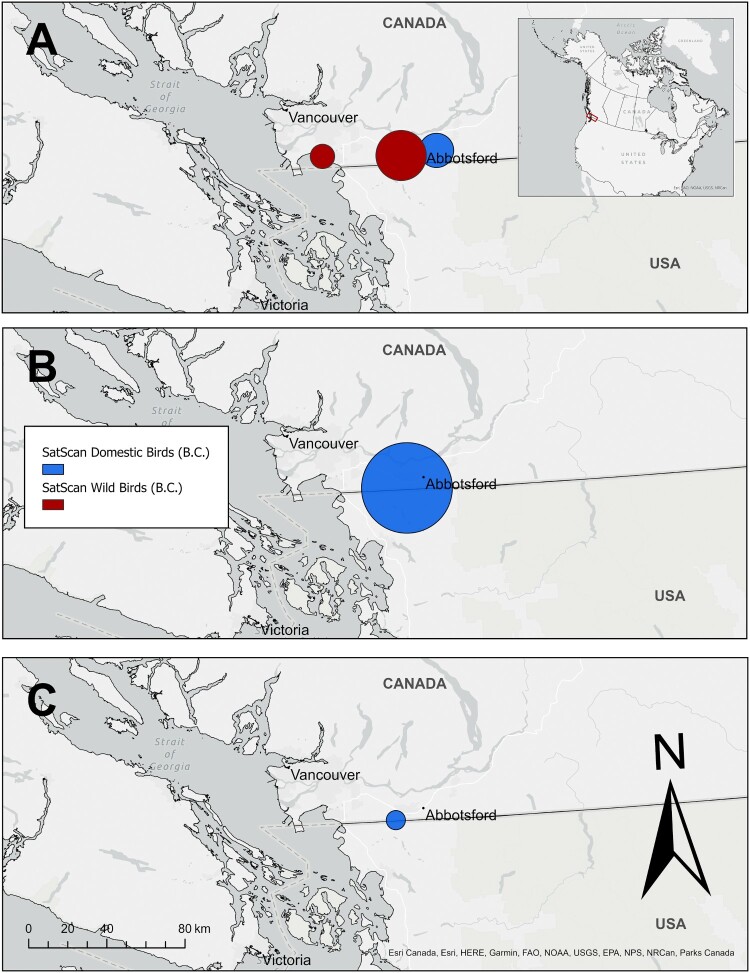
Locations of statistically significant (*p* < 0.05) spatial clusters of HPAI H5N1 clade 2.3.4.4b-positive wild birds (passive surveillance only, confirmed by whole genome sequencing) and poultry from British Columbia (B.C.) and the Yukon, Canada between September 12, 2022, and June 16, 2023. SaTScan™ analysis was performed on A) genetic Cluster 3, B) genetic Cluster 5 and C) genetic Cluster 6.

Although HPAI H5N1 detections occurred across a wide geographic area in B.C. and the Yukon (Figure 4), cases in poultry and wildlife were more concentrated within southwestern B.C. in “Wave 2” compared to “Wave 1” (Supplementary Figure 3). Furthermore, although the highest number of HPAI H5N1 detections occurred during November to December 2022 (Figure 1 and Figure 4B), detections during that period were less widely distributed than in the two months prior. This was particularly pronounced for poultry cases. Statistically significant (*p *< 0.05; Supplementary Table 3) spatial clustering was found for genetic Clusters 3, 5 and 6 (Figure 5). For genetic Cluster 3 there were two spatial clusters in wild birds and one in poultry, with limited overlap of the largest wild bird cluster and the domestic poultry cluster (Figure 5A). There was a single spatial cluster for poultry farms infected with genetic Cluster 5 and one for poultry farms infected with genetic Cluster 6 (Figure 5B, C), without any statistically significant spatial clustering of wild birds infected with either of these genetic clusters.

### Seven distinct HPAI H5N1 genotypes detected across two outbreak waves

**Figure 6.**
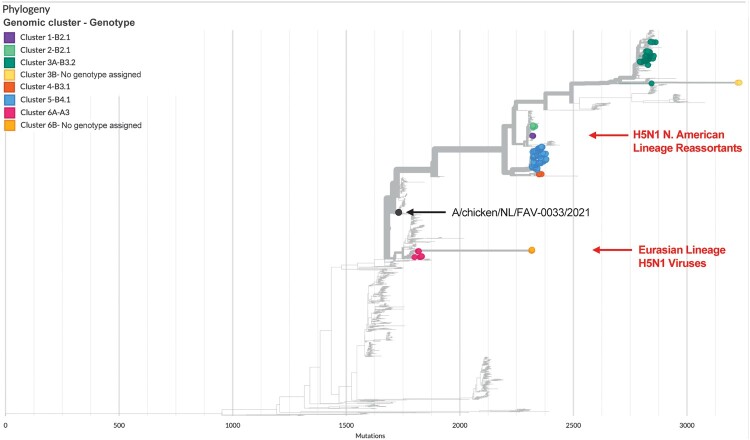
Concatenated full genome sequences of HPAI H5N1 clade 2.3.4.4b viruses from British Columbia (B.C.) and the Yukon, Canada from “Wave 1” and “Wave 2” contextualized with global H5N1 sequences to highlight genotypic diversity and viral reassortants. Built using a scaffold of global GISAID sequences, this tree displays HA-derived B.C./Yukon genetic clusters and their associated GenoFLU genotype classification, relative to the Eurasian (EA) A/chicken/NL/FAV-0033/2021 sequence detected at the start of the North American outbreak. Full genome tree is rooted by the A/Goose/Guangdong/1/96 (*Gs*/*Gd*) reference sequence.

Seven distinct HPAI H5N1 reassortment patterns or “genotypes” were detected in B.C. and the Yukon across the two waves of the outbreak, including four unique genotypes in “Wave 1” (Table 2, see Clusters 1, 2, 3A, 4 and 5) and three additional genotypes in “Wave 2” (Table 2, see Clusters 3B, 6A and 6B). These seven distinct genotypes were highlighted by constructing phylogeny from concatenated full genome sequences of these viruses, and contextualizing them with global H5N1 sequences collected during a similar timeframe (Figure 6). While all HPAI H5N1 viruses in “Wave 1” had at least two internal gene segments classified as North American (Am) lineage low pathogenicity avian influenza virus (LPAIV) reassortants (i.e. B2.1, B3.1, B3.2, B4.1 genotypes), viruses from “Wave 2” that were associated with Cluster 6A represented fully Eurasian (EA) lineage viruses (i.e. originating from Eurasia) belonging to the A3 genotype. Notably, EA segments associated with Clusters 1–5 represented a different EA genotype (EA1) than Cluster 6 viruses (EA3). Two additional groups of viruses (Cluster 3B and 6B) were not assigned a genotype because PA and PB1 segments did not meet minimum similarity thresholds to reference genotypes in the database, and their divergence from other Cluster 3 and Cluster 6 viruses was evident when full genome phylogeny from these viruses were compared (Figure 6). Cluster 3B viruses were assigned a different PB2 genotype (Am3.2) from 3A viruses, in addition to having two unassigned genotypes for PA and PB1 (Table 2). To understand the genetic differences between Cluster 6A and 6B viruses, pairwise SNP distances were used to explain why PA and PB1 segments lacked a genotype assignment. PA and PB1 segments had 10X more SNPs than the other segments (Supplementary Figure 4), suggesting that Cluster 6B viruses represent a novel genotype (at the time of writing, no closely related sequences were identified in global GISAID data). Cluster 6A viruses were only found in samples obtained from wildlife, while those belonging to Cluster 3B and 6B were only found in samples obtained from poultry (Table 2).

**Table 2.** Genotypic characterization and reassortant classification of HA-defined genetic clusters identified among “Wave 1” and “Wave 2” HPAI H5N1 viruses from British Columbia (B.C.) and the Yukon, Canada between April 2022 and June 2023. EA, Eurasian lineage virus segment; Am, North American lineage virus segment, as defined by GenoFLU reference database [21].

Genetic Cluster	Genotype by Influenza A Segment*^								GenoFLU Genotype^	Total wildlife samples (N (%))	Total poultry samples (N (%))
	HA	NA	M	NP	NS	PA	PB1	PB2			
**Cluster 1**	EA1	EA1	EA1	Am1.1	EA1	EA1	EA1	Am1.2	**B2**.**1**	0 (0%)	3 (3%)
**Cluster 2**	EA1	EA1	EA1	Am1.1	EA1	EA1	EA1	Am1.2	**B2**.**1**	1 (1%)	13 (11%)
**Cluster 3A**	EA1	EA1	EA1	Am1.4.1	Am1.1	EA1	Am1.2	Am2.1	**B3**.**2**	85 (45%)	25 (21%)
**Cluster 3B**	EA1	EA1	EA1	Am1.4.1	Am1.1	-	-	Am3.2	**Not assigned**	0 (0%)	2 (2%)
**Cluster 4**	EA1	EA1	EA1	Am1.4.1	EA1	EA1	EA1	Am1.2	**B3**.**1**	7 (4%)	0 (0%)
**Cluster 5**	EA1	EA1	EA1	Am1.3	EA1	EA1	EA1	Am2.2	**B4**.**1**	81 (42%)	65 (56%)
**Cluster 6A**	EA3	EA3	EA3	EA3	EA3	EA3	EA3	EA3	**A3**	17 (9%)	0 (0%)
**Cluster 6B**	EA3	EA3	EA3	EA3	EA3	-	-	EA3	**Not assigned**	0 (0%)	9 (8%)

*EA = Eurasian lineage; Am = North American lineage

^^^ Genotype assignment using GenoFlu tool (https://github.com/USDA-VS/GenoFLU); sequences identified as “Not assigned” indicate that no genotype was assigned to segments labelled as “-” due to no available database match with ≥ 98% identity. Nomenclature for GenoFLU Genotype: “A” denoted for unreassorted Eurasian lineage viruses, “B” for EA/Am reassortants.

Among the non-H5 AIV sequences (*N *= 12) detected through passive surveillance, one bird, a northern pintail (*Anas acuta*) collected on Jan. 1, 2023, was infected with a virus, subtyped as H12N8, for which the NP segment matched the Am1.3 NP segment detected among Cluster 5 sequences (Table 2).

## Discussion

Between April 2022 and June 2023, B.C. and the Yukon experienced two distinct “waves” of HPAI in wildlife and domestic poultry associated with H5N1 clade 2.3.4.4b, a descendant of the A/Goose/Guangdong/1/1996 H5 lineage. Compared to “Wave 1” (April – early September 2022), a greater number of wild animals and poultry farms were affected in the second wave (late September 2022 – January 2023), but cases were more concentrated in the southwest corner of B.C., particularly during the peak of the outbreak. While most of B.C. experiences a “classic” bi-modal increase in waterfowl density and diversity associated with birds migrating southwards in the fall and northwards in the spring, the mild climate of southwestern B.C., particularly the Fraser Valley, results in a great number of migratory waterfowl overwintering in this area [22]. The consequence of this being a peak in waterfowl density and diversity in fall and winter with an overall decline in those variables once birds depart in the spring [23]. Given that the Fraser Valley is also home to the highest density of poultry farms in Canada [24], the co-occurrence of large numbers of waterfowl and poultry within a small geographic area likely created the observed geotemporal HPAIV “hotspot” observed in this study.

During both waves, HPAI H5N1 infected wild birds were found throughout B.C. and the Yukon, suggesting a metapopulation model of HPAI spread [25,26]. It seems likely that migratory waterfowl are responsible for HPAIV introduction events, but subsequently, migratory and resident wild bird populations interact to quickly spread the virus in multiple directions [26].

Two broad categories of wild birds died from HPAI in “Wave 2” (Table 1, Supplementary Table 1). These included waterfowl (e.g. geese) who were likely infected by HPAIV through exposure to the virus in aquatic environments, and raptors/corvids, who were likely infected by consuming other HPAIV-infected birds [5,27]. While some raptors and corvids are recognized as scavengers (e.g. bald eagles and crows [28]) or active predators of waterfowl (e.g. peregrine falcon [28]), others (e.g. barn owls [28]) do not typically consume waterfowl; therefore, the origin of their infections is less clear.

The relative frequency of HPAIV-related mortalities in different wild bird species varied between the two waves, likely because of spatiotemporal variation in wild bird ecology and life history traits. For example, HPAIV-related mortalities occurred in cackling geese, swans, and lesser snow geese in the second wave but not the first, likely due to the fact that these species spend the summer in the far north, migrating through and/or overwintering in southern B.C., particularly to the Fraser Valley, during the fall and winter [23]. Differences in detection rates among species may also be attributable to differences in infection dynamics among the circulating HPAIV genotypes and/or differences in transmission patterns among different avian taxa or populations [29]. For example, the relatively high prevalence of infection in geese and swans may also be because these species are highly susceptible to HPAI H5N1 infection and thought to play an important role in the dispersal of the virus [30]. Furthermore, temporal variation in behaviour, and particularly food preferences, may also affect the likelihood of HPAIV infection. For example, there were fewer bald eagle mortalities in “Wave 2”, which may reflect the fact that bald eagles preferentially consume salmon in the fall and winter and may be more likely to scavenge HPAIV-infected birds in the spring and summer when salmon are not available [31].

As in “Wave 1”, certain waterfowl species traditionally considered to be HPAIV reservoirs (e.g. dabbling duck species such as mallard [*Anas platyrhynchos*], American wigeon [*Mareca americana*], and teal spp. [32]) were underrepresented in the passive surveillance data. It is possible that these species have a greater degree of adaptation or immunity to HPAI H5N1 and are not succumbing to infection [32,33]. In other studies [33], HPAIV-infected, apparently healthy mallards were detected through active surveillance. Similarly, previous field studies detected seroconversion to HPAI H5N1 in live mallards [32], and experimental HPAI H5N1 infection studies found that blue-winged teal (*Spatula discors*), mallard, redhead (*Aythya americana*), and northern pintail are refractory to disease [34]. This suggests that the utility of passive surveillance is highly dependent on host–pathogen relationships, and it may be necessary to add additional surveillance methods (e.g. active and/or environmental surveillance) to better detect HPAI H5N1 reservoirs.

Eight genetically distinct groups (or Clusters) of HPAI H5N1 were identified in B.C. and the Yukon over the two outbreak waves (Table 2). Although the majority of the viruses detected in the “Wave 2” were closely related to those detected in “Wave 1”, there was marked viral evolution over time, with some clusters subsiding and others becoming more established in “Wave 2” vs. “Wave 1”. For example, Clusters 1, 2, and 4 were identified in the first wave but not in the second, and detections of Cluster 3 increased dramatically to supersede Cluster 5 as the most dominant Cluster in “Wave 2”. These eight Clusters correspond with seven distinct genotypes (i.e. reassortment patterns), five of which were EA/Am reassortants of the A1 genotype, suggesting ongoing regional circulation of genotypes descendant from the original HPAI H5N1 introduction from Europe via Eastern Canada in 2021. With regard to the origin of the North American (i.e. “Am”) segments in these genotypes, a recent study found that they could be traced back to LPAIVs found in wild birds, suggesting that these reassortments occurred in wildlife as opposed to in poultry [35]. This is supported by our detection of a H12N8 AIV in a northern pintail that had an NP segment matching the Am1.3 NP segment detected among Cluster 5 sequences (Table 2).

Notably, Cluster 6 viruses, which were only detected in “Wave 2”, had 6–8 virus segments belonging to the A3 genotype, which is most closely related to a HPAI H5N1 strain circulating in East Asia, and likely entered North America in 2022 via birds traversing the East Asian-Australasian or West Pacific flyways prior to breeding in the Arctic along with birds from the Pacific flyway [36]. The convergence of birds from multiple flyways in the Arctic can result in spread of HPAI among those flyways, resulting in the infection of North American waterbirds who subsequently move the virus south during fall migration. This was also the mechanism for the introduction of HPAI H5N8 into North America in 2014 [37]. Interestingly, a Cluster 6A virus was detected in a single bald eagle in February 2022 [19], but then not again until November 2022. This may indicate two separate introduction events of this HPAI H5N1 virus from Asia to North America in 2022 [19].

During both waves of the outbreak, there were few patterns observed with regard to the distribution of the different genotypes in wild birds geographically, or with regard to the species infected, similar to what has been observed in other jurisdictions [26]. This suggests that HPAI H5N1 genotypes are not adapted to specific avian species and that the cosmopolitan host range allows for widespread distribution in association with the diverse movement patterns of the different species infected.

The mammalian species affected in B.C./Yukon included red foxes (*Vulpes vulpes*) and striped skunks in “Wave 1”, but only striped skunks in “Wave 2”. Although HPAI H5N1 infections have been detected in a variety of wild and domestic mammalian species, [38,39], skunks appear to be overrepresented among reported cases, consistent with what was observed in B.C. and the Yukon. This may suggest that skunks are more susceptible to HPAI H5N1 compared to other mammalian species – a hypothesis supported by previous instances where influenza viruses that originated in other species have infected skunks [40]. However, it is also possible that sick or dead skunks are easier to identify compared to other species because of their propensity towards living close to people, which is supported by the fact that most infected skunks in B.C. were found in major metropolitan areas.

Skunks and other mesocarnivores are thought to become infected with HPAI H5N1 by scavenging on infected dead wild birds [37]. As in previous studies of HPAI H5N1 in Canadian mesocarnivores [7], multiple HPAI H5N1 genotypes were detected in the infected skunks, all of which were related to viruses detected in wild birds, and infections in skunks occurred following the peak of wild bird detections. These findings support the assumption that viral spillover was most likely a result of the consumption of infected wild bird carcasses. However, there were several groups of infected skunks detected in close temporal and geographic proximity to one another that were infected by identical HPAI viruses. This may suggest that the animals were exposed to a common source; however, the possibility of lateral transmission among the skunks cannot be ruled out. It is also of note that the HPAI H5N1 viruses detected in the skunks had several unique mutations of potential significance for mammalian adaptation that were not detected in wild birds. It is unknown whether these mutations were circulating in wild birds but not detected or if they arose *de novo* within the skunk hosts.

Regarding spillover into poultry, the temporal peak in the number of infected farms aligned closely with the temporal peak in passive wild bird detections (Figure 1). This may suggest that increasing prevalence of HPAI H5N1 in wild birds increases the degree of environmental contamination and thus the risk of transmission to poultry, particularly in areas where there are both a high density of poultry farms and a high density and diversity of waterfowl [41], as is the case in southwestern B.C., and the Fraser Valley specifically. Conversely, there are few commercial-scale farms located in the Yukon, and waterfowl habitats are generally in areas untouched by human settlement. We found no clear alignment regarding the relative prevalence or geographic distribution of the HPAI H5N1 genotypes detected in both wild birds and poultry and some genotypes were detected only in wildlife (Cluster 4 in “Wave 1”, Cluster 6A in “Wave 2”), while others were detected only in poultry (Cluster 1 in “Wave 1”, Cluster 3A and 6B in “Wave 2”) (Table 2). Interestingly, there was significant spatial aggregation of genetic Clusters 5 and 6 in poultry but not in wild birds, and while spatial patterns in the distribution of Cluster 3 were detected in wild birds and poultry, those patterns overlapped only partially (Figure 5). If farm outbreaks are the result of independent incursions (i.e. wild bird to farm), one would expect that the prevalence and geospatial distribution of HPAI H5N1 genotypes in wild and domestic birds would be aligned. The fact that they were not might suggest a) gaps or biases in the wild bird surveillance and/or b) lateral transmission of HPAIV among poultry farms.

An important limitation of this study is its reliance on data from passive surveillance. As noted above, passive surveillance may not reflect the true prevalence, distribution, and host range of HPAIVs, particularly in wildlife reservoirs. Additionally, the absence of accurate denominator data for the wildlife populations being monitored presents a barrier to conducting more advanced epidemiological analyses. That being said, incorporating phylodynamic analyses in future studies would help to elucidate outbreak patterns by addressing correlational structures among data regarding host taxa, viral phylogeny, space, and time.

These data demonstrate that the ecology of HPAI changes rapidly over time in association with temporospatial variation in wild bird ecology. There was rapid evolution of the virus, including both reassortment and genetic drift, and viral genomics can help to further elucidate HPAIV epidemiology in wildlife and poultry, as well as virus transmission between the two. Similarly, concurrent epidemiological and viral sequence analyses provided evidence of HPAI H5N1 spillover into mesocarnivores from wild birds but could not rule out the possibility of lateral transmission or adaptive viral mutation among infected mammals.

## Supplementary Material

HPAI BC YT Wave 2_Supplementary Materials Updated Submission.docx

## Data Availability

The whole genome sequence data that support the findings of this study are publicly available on GISAID (https://www.gisaid.org/).
