# Supplementary material for: Descriptive epidemiology and phylogenetic analysis of highly pathogenic avian influenza H5N1 clade 2.3.4.4b in British Columbia (B.C.) and the Yukon, Canada, September 2022 to June 2023
Source: Emerg Microbes Infect. 2024 Aug 15;13(1):2392667. doi: 10.1080/22221751.2024.2392667 (PMC11421163; doi:10.1080/22221751.2024.2392667)
Supplement: HPAI BC YT Wave 2_Supplementary Materials Updated Submission.docx [file TEMI_A_2392667_SM1529.docx]

**Supplementary Materials**

**Descriptive epidemiology and phylogenetic analysis of highly pathogenic avian influenza H5N1 clade 2.3.4.4b in British Columbia (B.C.) and the Yukon, Canada, September 2022 to June 2023**

Shannon L. Russell^a,b^†, Cassandra L. Andrew^c^†*, Kevin C. Yang^a,d^, Michelle Coombe^e^, Glenna McGregor^e^, Tony Redford^e^, Agatha N. Jassem^a,b^, James E. A. Zlosnik^a,b^, Jolene Giacinti^f^, Kevin S. Kuchinski^a^, John Palmer^a^, John R. Tyson^a,b^, Chris Fjell^a,b^, Megan Willie^g^, Megan V. Ross^g^, Maeve Winchester^h^, Laurie Wilson^g^, Yohannes Berhane^i^, Caeley Thacker^h^, N. Jane Harms^j^, Catherine Soos^f^, Theresa Burns^e^, Natalie Prystajecky^a,b^, Chelsea Himsworth^c,e,k^

^a^ British Columbia Centre for Disease Control (BCCDC) Public Health Laboratory, Vancouver, Canada

^b^ Department of Pathology and Laboratory Medicine, University of British Columbia, Vancouver, Canada

^c^ School of Population and Public Health, Faculty of Medicine, University of British Columbia, Vancouver, Canada

^d^ Public Health Agency of Canada (PHAC), Winnipeg, Canada

^e^ Animal Health Centre, British Columbia Ministry of Agriculture and Food, Abbotsford, Canada

^f^ Ecotoxicology and Wildlife Health Division, Environment and Climate Change Canada (ECCC), Ottawa, Canada

^g^ Canadian Wildlife Service, Environment and Climate Change Canada (ECCC), Delta, Canada

^h^ British Columbia Ministry of Water, Land and Resource Stewardship, Nanaimo, Canada

^i^ Canadian Food Inspection Agency, Winnipeg, Canada

^j^ Department of Environment, Government of Yukon, Whitehorse, Canada

^k^ Canadian Wildlife Health Cooperative British Columbia, Abbotsford, Canada (C. Himsworth)

*****Dr. Cassandra Andrew, School of Population and Public Health, FacultyDepartment of Medicine, University of British Columbia, 2206 East Mall Vancouver, B.C., V6T 1Z3, Canada; email: [clandrew@student.ubc.ca](mailto:clandrew@student.ubc.ca); ORCID: <https://orcid.org/0000-0002-4925-4867>

† These first authors contributed equally to this article.

**Technical Appendix**

***Hunter harvest and lethal capture active surveillance***

Waterfowl specimens, including those from American wigeon (*Mareca americana*), green-winged teal (*Anas crecca*), northern shoveler (*Spatula clypeata*), northern pintail (*Anas* *acuta*), and lesser snow geese (*Anser caerulescens*) were collected at Vancouver International Airport, and the adjacent tidal foreshore of Sea Island, in Richmond, B.C. These animals were euthanized by airport wildlife management technicians on site and presumed to have been otherwise healthy. Euthanasia of waterfowl was carried out in conjunction with non-lethal dispersal techniques to reduce density and abundance of animals in and around the runway environment of the airport. These kill-to-scare activities were carried out under authorization of Migratory Bird Airport Permit AP-BC-2020-0001 and Migratory Bird Damage-Danger Permit DA-BC-2022-002. Animals were killed using steel shot (#BB) discharged from a 12-gauge shotgun. Collection of birds that were analyzed in this study occurred between November 20, 2022, and March 1, 2023. Additionally, a B.C. resident hunter contracted through CWS collected cloacal and choanal swabs from birds during their regular fall hunting activities under a B.C. Migratory Game Bird Hunting Permit.

***Live active wild bird surveillance***

Surf scoters (*Melanitta perspicillata*) and white-winged scoters (*Melanitta deglandi*) were captured using decoys and floating mist nets (1) in Boundary Bay, B.C. from November – December 2022. Activities were carried out under authorization of a Scientific Permit to Capture and Band Migratory Birds 10673X. Not all live captured birds were swabbed.

***R analyses***

Descriptive epidemiological analyses were conducted using R (v4.2.1 GUI 1.79 High Sierra build (8095)) (2) using the RStudio (v 2023.06.1+524) interface. The following packages were utilized: tibble (3), dplyr (4), stringr (5), tidyr (6), readr (7), readxl (8), lubridate (9), ggplot2 (10), scales (11), viridis (12), ISOweek (13), and here (14).

***SaTScan™ analysis***

SaTScan™ (v 10.1 2023) was used to identify geographic clustering of genetically related viruses in HPAI H5N1-positive poultry compared to wild birds. Only wild birds from the passive surveillance program and poultry cases from B.C. were included in the SaTScan™ analysis. Wild bird and poultry analyses were conducted separately, as was each genomic cluster (3, 5 or 6). For example, for wild birds, samples positive for ‘Cluster 3’ were ‘Cluster 3’ cases, and samples that were negative or any other cluster identification were controls. This process was repeated for each cluster. The same process was used for domestic poultry premises, except that there were no negative farms included in the analyses. We used a purely spatial, Bernoulli probability model with a circular scanning statistic, scanning for both high and low rates (15,16).

***Phylogenetic trees***

Hemagglutinin (HA)-specific phylogenetic trees contextualized with sequences from the Americas contained sequences from B.C. and the Yukon, as well as publicly available H5N1 sequences from Canada (*n*=80), the U.S.A. (*n*=1171), Central America (*n*=6) and South America (*n*=34) were downloaded from the Global Initiative on Sharing All Influenza Data (GISAID) database (<https://www.gisaid.org/> (17)) with specimen collection dates between September 1, 2021 and July 15, 2023 (EPI_ISL IDs are available upon request). For poultry (one sequence per infected premise) and mammal-specific genomic analyses, HA phylogeny consisting of only B.C. and Yukon sequences were constructed, rooted on the fully Eurasian H5N1 virus introduced in Newfoundland (NL) in December 2021 (A/chicken/NL/FAV0033/2021). Phylogenetic trees were constructed using IQ-Tree (19) in Augur (20) and visualized in Nextstrain (21). Sequences were confirmed to be H5 clade 2.3.4.4b using the Nextclade (22) clade-calling tool paired with a custom H5NX reference dataset built in-house (https://github.com/BCCDC-PHL/nextclade-generator).

Reassortant H5N1 viruses from ‘Wave 1’ and ‘Wave 2’ datasets were identified by genotype classification using the GenoFLU tool (23; <https://github.com/USDA-VS/GenoFLU>), which assigns a genotype to each segment . Genotype assignments were confirmed by constructing phylogeny for each gene segment (HA, matrix (M), neuraminidase (NA), nucleoprotein (NP), non-structural proteins (NS), polymerase acidic (PA), polymerase basic 1 (PB1), polymerase basic 2 (PB2)) comprised of local (B.C./Yukon) and global GISAID H5N1 sequences collected between September 15, 2021–July 15, 2023 (EPI_ISL IDs are available upon request). In order to visualize the diverse genome constellations present in ‘Wave 1’ and ‘Wave 2’ datasets, concatenated full genome sequences (requiring a minimum 20X depth and 90% coverage across each segment) were incorporated into a phylogenetic tree with global GISAID sequences described above. Segments from local and publicly available global data were concatenated into contiguous full genome sequences using the same lab-developed concatenation script. Our group is aware that the concatenation of influenza segments, which are subject to reassortment, is at odds with the core phylogenetic assumption of no recombination. However, our awareness of this caveat and use of these trees for high-level visualization only justifies their use from a practicality standpoint. In all phylogenetic analyses, sequences were defined by the date the sample was collected.

***Mammalian adaptive mutational scan***

Frequencies of mammalian adaptive mutations in wildlife and poultry sequences were quantified by scanning sequences incorporated into segment-specific (i.e., HA, M, NS, NP, PA, PB1 and PB2) phylogenetic trees for mutations of interest using the Nextstrain-produced aa_muts.json file.

***Supplementary Tables***

**Supplementary Table 1.** Percent positivity for HPAI H5 by polymerase chain reaction (PCR) in wild species (birds and mammals) collected through both passive and active surveillance programs in British Columbia (B.C.) and the Yukon Territory (YT) between September 12, 2022, and June 16, 2023

| Species | | Percent positivity (%) |
| --- | --- | --- |
| *Active hunter harvested or lethal capture bird surveillance – British Columbia (B.C.) only* | | |
| Lesser snow goose (*Anser caerulescens*) | | 2/7 (29) |
| Mallard (*Anas platyrhynchos*) | | 7/50 (14) |
| American wigeon (*Mareca americana)* | | 1/20 (5) |
| Green-winged teal (*Anas crecca*) | | 0/4 (0) |
| Northern pintail (*Anas acuta*) | | 0/4 (0)^a^ |
| Northern shoveler (*Spatula clypeata*) | | 0/2 (0) |
|  | | 10/87 (12) |
| *Active live bird surveillance – British Columbia (B.C.) only* | | |
| White-winged scoter (*Melanitta deglandi*) | | 0/34 (0) |
| Surf scoter (*Melanitta perspicillata)* | | 0/16 (0) |
|  | | 1/50 (0) |
| *Passive bird surveillance – British Columbia (B.C.)* | | |
| Peregrine falcon (*Falco peregrinus*) | | 6/6 (100) |
| Dunlin (*Calidris alpina*) | | 2/2 (100) |
| American white pelican (*Pelecanus erythrorhynchos*) | | 1/1 (100) |
| Barrow's goldeneye (*Bucephala islandica*) | | 1/1 (100) |
| Brant (*Branta bernicla*) | | 1/1 (100) |
| Common goldeneye (*Bucephala clangula*) | | 1/1 (100) |
| Turkey vulture (*Cathartes aura*) | | 1/1 (100) |
| Red-tailed hawk (*Buteo jamaicensis*) | | 13/16 (81) |
| Cackling goose (*Branta hutchinsii*) | | 7/10 (70) |
| Barn owl (*Tyto alba*) | | 4/6 (67) |
| American wigeon (*Mareca americana*) | | 2/3 (67) |
| Canada goose (*Branta canadensis*) | | 15/23 (65) |
| Lesser snow goose (*Anser caerulescens*) | | 12/19 (63) |
| American crow (*Corvus brachyrhynchos*) | | 16/32 (50) |
| Trumpeter swan (*Cygnus buccinator*) | | 15/30 (50) |
| Long-eared owl (*Asio otus*) | | 1/2 (50) |
| Wood duck (*Aix sponsa*) | | 1/2 (50) |
| Great horned owl (*Bubo virginianus*) | | 12/27 (44) |
| Cooper's hawk (*Accipiter cooperii*) | | 4/10 (40) |
| Great blue heron (*Ardea herodias*) | | 3/9 (33) |
| Tundra swan (*Cygnus columbianus*) | | 1/3 (33) |
| Bald eagle (*Haliaeetus leucocephalus*) | | 6/25 (24) |
| Barred owl (*Strix varia*) | | 3/16 (19) |
| Common raven (*Corvus corax*) | | 1/6 (17) |
| Glaucous-winged gull (*Larus glaucescens*) | | 4/32 (13) |
| American coot (*Fulica americana)* | | 0/2 (0) |
| American robin (*Turdus migratorius*) | | 0/3 (0) |
| Ancient murrelet (*Synthliboramphus antiquus*) | | 0/1 (0) |
| Anna's hummingbird (*Calypte anna*) | | 0/2 (0) |
| Black-capped chickadee (*Poecile atricapillus*) | | 0/1 (0) |
| Bohemian waxwing (*Bombycilla garrulus*) | | 0/4 (0) |
| Boreal owl (*Aegolius funereus*) | | 0/1 (0) |
| Bufflehead (*Bucephala albeola*) | | 0/1 (0) |
| California quail (*Callipepla californica*) | | 0/2 (0) |
| Cedar waxwing (*Bombycilla cedrorum*) | | 0/2 (0) |
| Common merganser (*Mergus merganser*) | | 0/1 (0) |
| Common murre (*Uria aalge*) | | 0/1 (0) |
| Dark-eyed junco (*Junco hyemalis*) | | 0/2 (0) |
| Double-crested cormorant (*Nannopterum auritum*) | | 0/1 (0) |
| Eurasian collared dove (*Streptopelia decaoctoI*) | | 0/2 (0) |
| Golden eagle (*Aquila chrysaetos*) | | 0/1 (0) |
| Golden-crowned sparrow (*Zonotrichia atricapilla*) | | 0/1 (0) |
| Hooded merganser (*Lophodytes cucullatus*) | | 0/1 (0) |
| House finch (*Haemorhous mexicanus*) | | 0/1 (0) |
| Lesser scaup (*Aythya affinis*) | | 0/1 (0) |
| Mallard (*Anas platyrhynchos*) | | 0/10 (0) |
| Merlin (*Falco columbarius*) | | 0/4 (0) |
| Mourning dove (*Zenaida macroura*) | | 0/9 (0) |
| Northern flicker (*Colaptes auratus*) | | 0/2 (0) |
| Northern pintail (*Anas acuta*) | | 0/1 (0) |
| Northern saw-whet owl (*Aegolius acadicus*) | | 0/7 (0) |
| Pacific loon (*Gavia pacifica*) | | 0/1 (0) |
| Pine siskin (*Spinus pinus*) | | 0/2 (0) |
| Red-breasted merganser (*Mergus serrator*) | | 0/1 (0) |
| Ring-necked pheasant (*Phasianus colchicus*) | | 0/1 (0) |
| Rock pigeon (*Columba livia*) | | 0/3 (0) |
| Ruby-crowned kinglet (*Corthylio calendula*) | | 0/1 (0) |
| Ruddy duck (*Oxyura jamaicensis*) | | 0/1 (0) |
| Ruffed grouse (*Bonasa umbellus*) | | 0/2 (0) |
| Rufous hummingbird (*Selasphorus rufus*) | | 0/1 (0) |
| Sharp-shinned hawk (*Accipiter striatus*) | | 0/8 (0) |
| Short-eared owl (*Asio flammeus*) | | 0/1 (0) |
| Song sparrow (*Melospiza melodia*) | | 0/1 (0) |
| Spotted towhee (*Pipilo maculatus*) | | 0/3 (0) |
| Steller's jay (*Cyanocitta stelleri*) | | 0/3 (0) |
| Varied thrush (*Ixoreus naevius*) | | 0/16 (0) |
| Virginia rail (*Rallus limicola*) | | 0/1 (0) |
|  | | 134/394 (34) |
| *Passive bird surveillance – Yukon Territory (YT)* | | |
| Bald eagle (*Haliaeetus leucocephalus*) | | 1/3 (33) |
| Common raven (*Corvus corax*) | | 1/3 (33) |
| Bohemian waxwing (*Bombycilla garrulus*) | | 0/2 (0) |
| Boreal owl (*Aegolius funereus*) | | 0/2 (0) |
| Common redpoll (*Acanthis flammea*) | | 0/1 (0) |
| Great horned owl (*Bubo virginianus*) | | 0/1 (0) |
| Merlin (*Falco columbarius*) | | 0/2 (0) |
| Pacific loon (*Gavia pacifica*) | | 0/1 (0) |
| Red crossbill (*Loxia curvirostra*) | | 0/2 (0) |
| Sharp-shinned hawk (*Accipiter striatus*) | | 0/1 (0) |
| Spruce grouse (*Falcipennis canadensis*) | | 0/1 (0) |
| Trumpeter swan (*Cygnus buccinator*) | | 0/2 (0) |
| Varied thrush (*Ixoreus naevius*) | | 0/1 (0) |
| White-winged crossbill (*Loxia leucoptera*) | | 0/1 (0) |
|  | | 2/23 (9) |
| *Passive mammal surveillance – British Columbia (B.C.) and Yukon Territory (YT)* | | |
| Striped skunk (*Mephitis mephitis*) – B.C. | 14/14 (100) | |
| American mink (*Mustela vison*) – B.C. | 0/1 (0) | |
| Muskrat (*Ondatra zibethicus*) – B.C. | 0/1 (0) | |
| Raccoon (*Procyon lotor*) – B.C. | 0/1 (0) | |
| American marten (*Martes americana*) – YT | 0/1 (0) | |
| Coyote (*Canis latrans*) – YT | 0/2 (0) | |
| Short-tailed weasel (*Mustela erminea*) – YT | 0/1 (0) | |
|  | 14/21 (67) | |

**Supplementary Table 2.** Frequency of mammalian adaptive mutations in HPAI H5N1 sequences from wild/domestic avian species and skunks during ‘waves’ 1 and 2 of the B.C./Yukon outbreak (adapted from (18)). Bold font indicates mutations that were differentially detected in avian and skunk sequences.

| Gene | | Mutation  (H5 Numbering) | Presence in B.C./Yukon avian sequences^a^  (N<374^b^, between 2022-2023) | Presence in B.C. skunks^a^  (N<12^b^, 2023) |
| --- | --- | --- | --- | --- |
| PB2 | | T271A | 0% | 0% |
|  |  | K526R | 0% | 0% |
|  |  | **E627K/E627A** | 0% | **11% / 33%** |
|  |  | **D701N** | 0% | **56%** |
|  |  | S714R | 0% | 0% |
| PB1 | | D3V | 100% | 100% |
|  |  | N105S | 0% | 0% |
|  |  | D622G | 100% | 100% |
| PA | | S37A | 100% | 100% |
|  |  | V63I | 0% | 0% |
|  |  | **K356R** | **1.5%** | **0%** |
|  |  | **N383D** | **99%** | **100%** |
|  |  | **N409S** | **99%** | **100%** |
| HA | | D94S | 100% | 100% |
|  |  | S133A | 100% | 100% |
|  |  | S154N | 100% | 100% |
|  |  | S155D | 100% | 100% |
|  |  | T156A | 100% | 100% |
|  |  | N182K | 0% | 0% |
|  |  | **T188I** | **4%** | **0%** |
|  |  | K189N | 100% | 100% |
|  |  | **V210A** | **92%** | **42%** |
|  |  | K218Q | 100% | 100% |
|  |  | Q222L | 0% | 0% |
|  |  | S223R | 100% | 100% |
|  |  | G224S | 0% | 0% |
| NP | | **N319K** | **6%** | **58%** |
|  |  | E434K | 0% | 0% |
| M | | I43M | 100% | 100% |
|  |  | T215A | 100% | 100% |
| NS | NS | 80-84DEL | 0% | 0% |
|  |  | D92E | 0% | 0% |
|  |  | I106M | 100% | 100% |
|  |  | C138F | 100% | 100% |
|  |  | N30D | 100% | 100% |

^a^ Mutations in neuraminidase were not included here.

^b^ Number of sequences analyzed per segment differs due to differences in segment coverage.

**Supplementary Table 3.** SaTScan™ geographic cluster statistical results for each genomic cluster (3-6) and bird type (wild or domestic) using Bernoulli spatial scan.

| Genomic cluster and classification (wild or domestic) | SaTScan™ Cluster | Expected cases | Observed/expected cases | Relative risk | p-value |
| --- | --- | --- | --- | --- | --- |
| Cluster 3 wild birds | 1 | 5.67 | 2.82 | 3.21 | 0.011^a^ |
|  | 2 | 1.31 | 4.58 | 4.84 | 0.042^a^ |
|  | 3 | 3.06 | 3.27 | 3.55 | 0.084 |
|  | 4 | 9.82 | 0.10 | 0.092 | 0.086 |
|  | 5 | 9.17 | 0.11 | 0.099 | 0.189 |
|  | 6 | 1.53 | 3.93 | 4.13 | 0.299 |
|  | 7 | 1.53 | 3.93 | 4.13 | 0.299 |
|  | 8 | 5.24 | 0 | 0 | 0.537 |
|  | 9 | 3.93 | 2.55 | 2.74 | 0.766 |
|  | 10 | 4.36 | 0 | 0 | 0.792 |
|  | 11 | 4.36 | 0 | 0 | 0.792 |
|  | 12 | 5.24 | 0.19 | 0.18 | 0.999 |
| Cluster 3 domestic poultry | 1 | 0.95 | 14.73 | 110.81 | <0.001^a^ |
| Cluster 5 wild birds | 1 | 0.12 | 16.68 | 18.04 | 0.284 |
|  | 2 | 0.12 | 16.68 | 18.04 | 0.585 |
|  | 3 | 0.12 | 16.68 | 18.04 | 0.585 |
|  | 4 | 0.90 | 5.56 | 6.70 | 0.602 |
|  | 5 | 4.44 | 0 | 0 | 0.670 |
|  | 6 | 0.24 | 8.34 | 8.98 | 0.994 |
|  | 7 | 0.30 | 6.67 | 7.17 | 0.994 |
|  | 8 | 2.16 | 0 | 0 | 0.994 |
| Cluster 5 domestic poultry | 1 | 10.18 | 3.63 | 28.01 | <0.001^a^ |
|  | 2 | 0.065 | 30.65 | 32.17 | 0.861 |
| Cluster 6 wild birds | 1 | 0.058 | 34.75 | 41.50 | 0.081 |
|  | 2 | 3.57 | 0 | 0 | 0.725 |
|  | 3 | 0.14 | 13.90 | 16.48 | 0.819 |
|  | 4 | 0.14 | 13.90 | 16.48 | 0.819 |
|  | 5 | 0.69 | 4.34 | 5.46 | 0.997 |
| Cluster 6 domestic poultry | 1 | 0.39 | 22.99 | Infinity | <0.001^a^ |

^a^ Statistically significant result with alpha = 0.05

**Supplementary Figures**

**
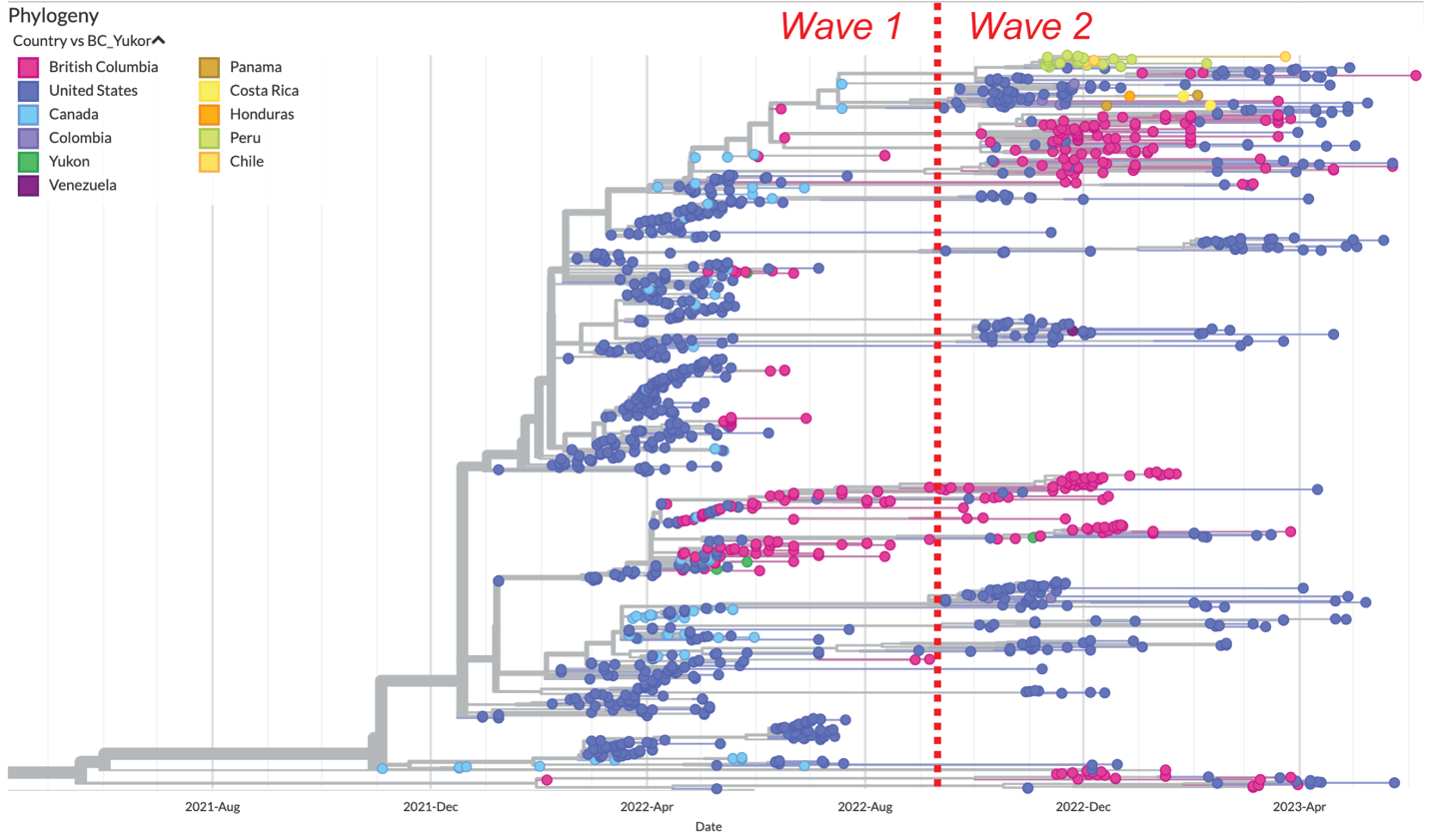
**

**Supplementary Figure 1.** A hemagglutinin (HA)-specific phylogenetic tree of H5N1 cases detected in British Columbia (B.C.) and the Yukon contextualized by additional H5N1 sequences from other parts of North and South America between September 2021 and July 2023. Colors denote each sequence’s country of origin. Specimen collection dates were used to scale this tree in time, such that branch lengths represent time instead of genetic distance. Trees are rooted by the A/Goose/Guangdong/1/96 (*Gs*/*Gd*) (H5) reference sequence.

**
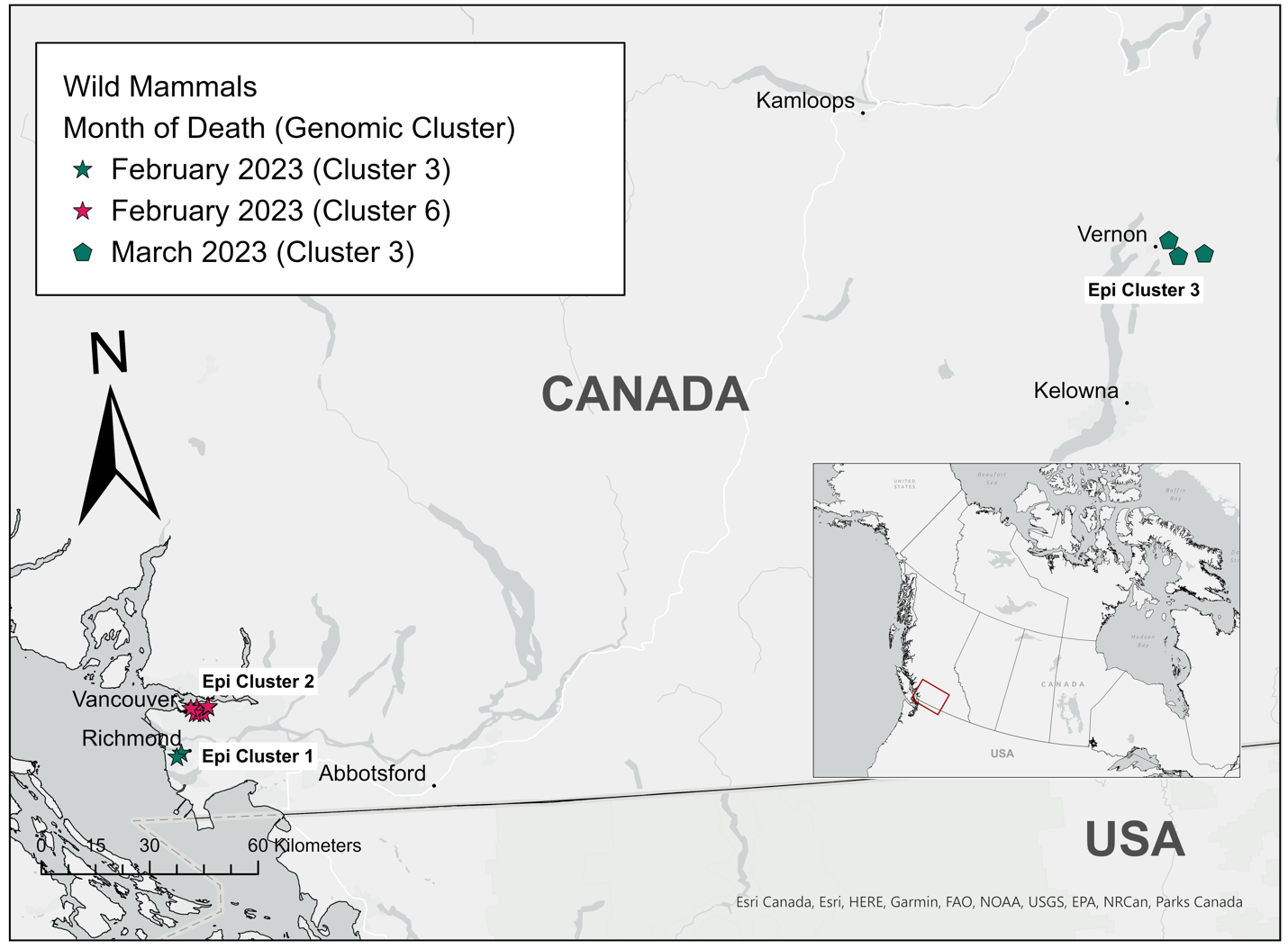
**

**Supplementary Figure 2.** Temporospatial distribution of HPAI H5N1 genetic clusters of striped skunk (*Mephitis mephitis*) samples, highlighting locations of detections and month of detection. Symbols indicate month detected (February, star; March, hexagon) and colours indicate genomic cluster identification (Cluster 3, green; Cluster 6, pink), with three discrete temporospatial ‘Epi Clusters’ identified.


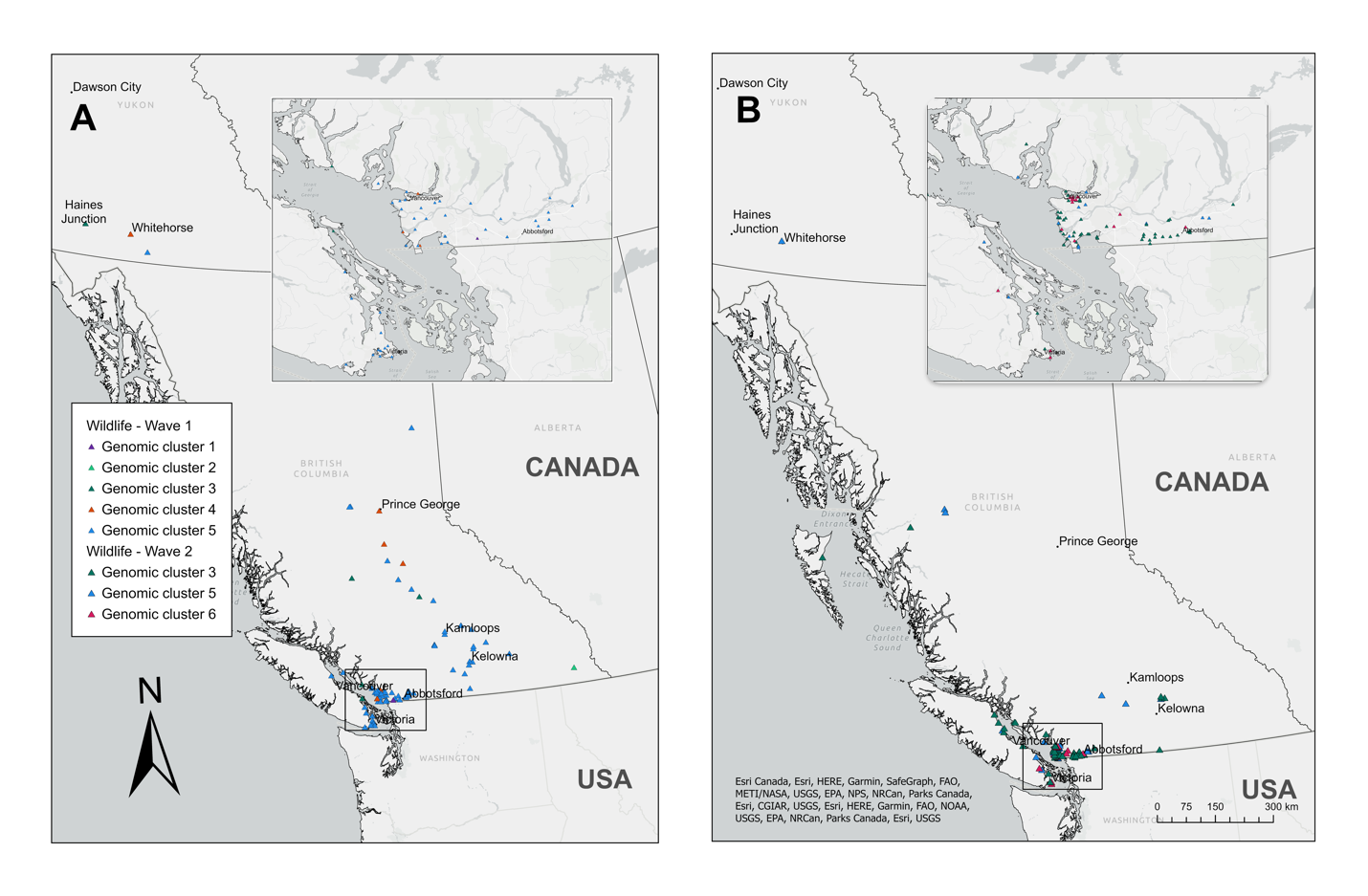


**Supplementary Figure 3.** Comparison of wild animal (avian and mammalian) cases and genetic cluster identifications of HPAI H5N1 2.3.4.4b detected between A) ‘Wave 1’ (April 12, 2022, to September 11, 2022), and B) ‘Wave 2’ (September 12, 2022, to June 16, 2023) in British Columbia (B.C.) and the Yukon, Canada

**
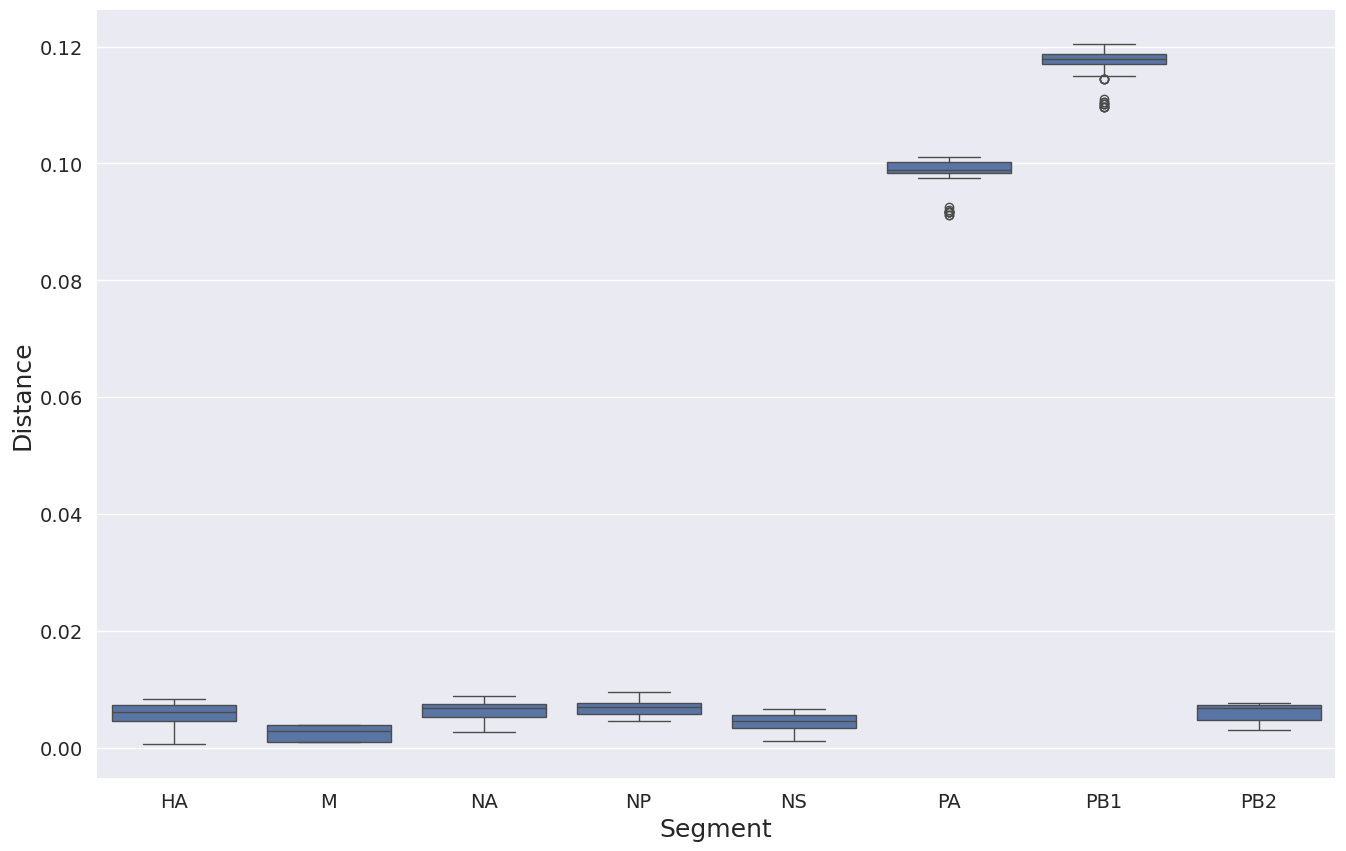
**

**Supplementary Figure 4.** Pairwise single nucleotide polymorphisms (SNP) distances between Cluster 6A and 6B on a per-segment basis. For each segment, distances were computed between all Cluster 6A sequences and all Cluster 6B sequences to produce a distribution. Distances are measured based on the average number of SNPs per site.

**Supplementary References**

1. Brodeur S, Mittelhauser GH, Savard JPL, Thomas PW, Titman RD, Comeau D. Capture Methods for Migrating, Wintering and Molting Sea Ducks. Waterbirds: The International Journal of Waterbird Biology. 2008;31:133–7.

2. R Core Team. R: A Language and Environment for Statistical Computing [Internet]. Vienna, Austria: R Foundation for Statistical Computing; 2022. Available from: https://www.R-project.org/

3. Müller K, Wickham H. tibble: Simple Data Frames [Internet]. 2023. Available from: https://CRAN.R-project.org/package=tibble

4. Wickham H, François R, Henry L, Müller K, Vaughan D. dplyr: A Grammar of Data Manipulation [Internet]. 2023. Available from: https://CRAN.R-project.org/package=dplyr

5. Wickham H. stringr: Simple, Consistent Wrappers for Common String Operations [Internet]. 2022. Available from: https://CRAN.R-project.org/package=stringr

6. Wickham H, Vaughan D, Girlich M. tidyr: Tidy Messy Data [Internet]. 2023. Available from: https://CRAN.R-project.org/package=tidyr

7. Wickham H, Hester J, Bryan J. readr: Read Rectangular Text Data [Internet]. 2023. Available from: https://CRAN.R-project.org/package=readr

8. Wickham H, Bryan J. readxl: Read Excel Files [Internet]. 2023. Available from: https://CRAN.R-project.org/package=readxl

9. Grolemund G, Wickham H. Dates and Times Made Easy with lubridate. Journal of Statistical Software. 2011;40(3):1–25.

10. Wickham H. ggplot2: Elegant Graphics for Data Analysis [Internet]. Springer-Verlag New York; 2016. Available from: https://ggplot2.tidyverse.org

11. Wickham H, Seidel D. scales: Scale Functions for Visualization [Internet]. 2022. Available from: https://CRAN.R-project.org/package=scales

12. Garnier, Simon, Ross, Noam, Rudis, Robert, et al. viridis - Colorblind-Friendly Color Maps for R [Internet]. 2021. Available from: https://sjmgarnier.github.io/viridis/

13. Block U, Hatzfeld using an algorithm by H von. ISOweek: Week of the year and weekday according to ISO 8601 [Internet]. 2011. Available from: https://CRAN.R-project.org/package=ISOweek

14. Müller K. here: A Simpler Way to Find Your Files [Internet]. 2020. Available from: https://CRAN.R-project.org/package=here

15. Kulldorff M. A spatial scan statistic. Communications in Statistics - Theory and Methods. 1997 Jan;26(6):1481–96.

16. Kulldorff, M. SaTScan Software for the spatial and space-time scan statistics [Internet]. Harvard Medical School, Boston and Information Management Services Inc.; 2023. Available from: http://www.satscan.org/

17. Khare S, Gurry C, Freitas L, B Schultz M, Bach G, Diallo A, et al. GISAID’s Role in Pandemic Response. China CDC Weekly. 2021;3(49):1049–51.

18. Skowronski, DM, Montoya S, Kaweski S, Ahmed B, Kim S, Jassem AN, et al. Risk assessment: human health implications of the clade 2.3.4.4b highly pathogenic avian influenza (HPAI) H5Nx epizootic, 2021-2022 [Internet]. British Columbia (BC) Centre for Disease Control (BCCDC); 2022 May [cited 2023 Dec 16]. Available from: <http://www.bccdc.ca/resource-gallery/Documents/Statistics%20and%20Research/Statistics%20and%20Reports/Epid/Influenza%20and%20Respiratory/ERV/HPAI_H5N1_Risk_Assessment_19_May_2022.pdf>

19. Huddleston J, Hadfield J, Sibley TR, Lee J, Fay K, Ilcisin M, Harkins E, Bedford T, Neher RA, Hodcroft EB. Augur: a bioinformatics toolkit for phylogenetic analyses of human pathogens. J Open Source Softw. 2021;6(57):2906. doi: 10.21105/joss.02906. Epub 2021 Jan 7. PMID: 34189396; PMCID: PMC8237802.

20. Minh, Bui Quang, et al. "IQ-TREE 2: new models and efficient methods for phylogenetic inference in the genomic era." *Molecular biology and evolution* 37.5 (2020): 1530-1534.

21. Hadfield, James, et al. "Nextstrain: real-time tracking of pathogen evolution." *Bioinformatics* 34.23 (2018): 4121-4123.

22. Aksamentov, Ivan, et al. "Nextclade: clade assignment, mutation calling and quality control for viral genomes." *Journal of open source software* 6.67 (2021): 3773.

23. Youk, Sungsu, et al. "H5N1 highly pathogenic avian influenza clade 2.3. 4.4 b in wild and domestic birds: Introductions into the United States and reassortments, December 2021–April 2022." *Virology* 587 (2023): 109860.
